# Telomere-to-Telomere Genome Assembly of Two *Hemiculter* Species Provide Insights into the Genomic and Morphometric Bases of Adaptation to Flow Velocity

**DOI:** 10.3390/biom16010083

**Published:** 2026-01-04

**Authors:** Jie Liu, Denghua Yin, Fengjiao Ma, Min Jiang, Xinyue Wang, Pan Wang, Kai Liu

**Affiliations:** 1 Key Laboratory of Freshwater Fisheries and Germplasm Resources Utilization, Ministry of Agriculture and Rural Affairs, Freshwater Fisheries Research Center, Chinese Academy of Fishery Sciences, Wuxi 214081, China; maydayjiel@163.com (J.L.);; 2 School of Ecology and Environment, Anhui Normal University, Wuhu 241000, China; 3 Wuxi Fisheries College, Nanjing Agricultural University, Wuxi 214081, China

**Keywords:** flow velocity, telomere-to-telomere, genome assembly, gap-free, morphometrics, *Hemiculter bleekeri*, *Hemiculter leucisculus*

## Abstract

Flow velocity is a key environmental factor that exerts multifaceted effects on fish growth and adaptation. Through long-term natural selection, fish have evolved adaptability to specific flow conditions, which not only relate to oxygen supply and food acquisition but also play a decisive role in reproduction, development, and population maintenance. To investigate the genomic mechanisms through which hydrodynamic environments drive divergence in closely related species, we focused on two sister species, *Hemiculter bleekeri* and *Hemiculter leucisculus*, which are adapted to contrasting flow regimes. We generated high-quality, chromosome level telomere-to-telomere (T2T) genomes and integrated comparative genomic analyses, we investigated the genetic basis underlying body shape regulation and reproductive strategies, aiming to decipher the adaptive evolutionary patterns of these species in response to differing hydrodynamic conditions from an integrated genotype phenotype perspective. We integrated PacBio HiFi, Hi-C, and Oxford Nanopore Technologies (ONT) ultra-long read sequencing data to construct high-quality T2T reference genomes for both species. The final genome assemblies are 0.998 Gb for *H. bleekeri* and 1.05 Gb for *H. leucisculus*, with each species possessing 24 chromosomes and all chromosomal sequences assembled into single contigs. Contig N50 values reached 40.45 Mb and 40.66 Mb, respectively, and both assemblies are gap-free. BUSCO assessments yielded completeness scores of 99.34% for both genomes, confirming their high continuity and accuracy. Integrated morphometric and genomic analyses revealed distinct adaptive strategies in two *Hemiculter* Species. *H. bleekeri* has evolved a streamlined body, underpinned by expansions in body shape related genes, and a pelagic egg strategy. In contrast, the adhesive egg strategy of *H. leucisculus* is supported by expansions in adhesion-related gene families. This divergence reflects adaptation to distinct flow velocity. By combining high-quality chromosome-level T2T genomes with morphometric and comparative genomic approaches, this study establishes a comprehensive framework for understanding the molecular mechanisms underlying adaptive evolution in freshwater fishes inhabiting contrasting flow velocity.

## 1. Introduction

Fish in natural aquatic environments are continuously interacting with hydrodynamic conditions. Flow velocity, as a key environmental factors, exerts multifaceted and complex effects on fish growth [1,2]. Fish generally select habitats that offer optimal flow velocities and abundant food resources to maximize energetic efficiency [3]. Through long-term natural selection, fish have evolved adaptability to specific flow conditions, which not only relate to oxygen supply and food acquisition but also play a decisive role in reproduction, development, and population maintenance [4].

This study focuses on a pair of sister species within the genus *Hemiculter* of the family Cyprinidae, *Hemiculter bleekeri* and *Hemiculter leucisculus*. Both species are widely distributed across major river systems from northern to southern China, and their population dynamics are primarily shaped by natural environmental conditions and genetic factors, with minimal influence from anthropogenic activities such as artificial stocking. Consequently, they serve as ideal model organisms for investigating ecological adaptation strategies [5]. Although they are highly similar in morphology and diet, they differ in habitat selection and reproductive strategies [6]. *H. bleekeri* predominates in high-velocity flow habitats, such as the main channel of the Yangtze River, whereas *H. leucisculus* is widely distributed across diverse environments, including slow-flowing and lentic water bodies. Their differential adaptation to flow velocity provides a valuable model system for exploring ecological divergence and adaptive mechanisms among closely related species [7,8,9].

To adapt to distinct hydrodynamic environments, *H. bleekeri* and *H. leucisculus* have evolved notable differences in morphology and reproductive strategies. Morphologically, the lateral line systems of the two species exhibit significant divergence, serving as a reliable diagnostic characteristic for rapid identification. In *H. bleekeri*, the lateral line of descends from the head in a smooth, deeply arched curve without pronounced angulation, comprising 47–52 pored scales; while *H. leucisculus* displays a conspicuous bend of approximately 120–135° posterior to the pectoral fin, with 40–47 lateral line scales [10]. These morphological differences may be functionally associated with differential capabilities in hydrodynamic sensing and spatial positioning under varying flow velocity environments. Additionally, *H. bleekeri* mainly inhabits high velocity, turbid open waters and holds a dominant position in the main channel of the Yangtze River [11]. Its spawning period coincides with the flood season of the Yangtze River, and it lays pelagic eggs to adapt to high-flowing conditions. Conversely, *H. leucisculus* typically spawns in shallow slow- flowing or lentic habitats, attaching its adhesive eggs to substrates such as gravel, sand, or aquatic plants. These contrasting traits reflect ecological differentiation in response to flow regime preferences and represent a key mechanism facilitating their coexistence within overlapping distribution ranges.

Currently, research on *H. bleekeri* and *H. leucisculus* mainly focuses on population ecology and reproductive biology, whereas studies in molecular genetics remain relatively limited. Existing efforts have largely been confined to mitochondrial whole-genome sequencing [12,13,14] and analyses of population genetic diversity [15,16]. The lack of high quality chromosome-level genomic data has hindered comprehensive investigations into the molecular mechanisms underlying ecological adaptation. In recent years, advancements in long-read sequencing technologies such as PacBio HiFi and Oxford Nanopore Technologies (ONT) Ultra-long, combined with Hi-C chromosome conformation capture techniques, have enabled telomere-to-telomere (T2T) gap-free genome assembly [17]. These technological breakthroughs now enable the construction of highly accurate and contiguous reference genomes for fish species [18]. Such complete genomes allow for complete characterization of previously inaccessible regions, including centromeres and telomeres, accurately identifying large-scale insertions/deletions and inversions related to ecological adaptability. and supporting genome-wide positive selection analysis and conservation comparison. Furthermore, such advances provide a robust foundation for elucidating the molecular basis of environmental adaptability within the genus *Hemiculter*.

To elucidate the genomic mechanisms underlying the adaptation of *H. bleekeri* and *H. leucisculus* to varying water flow velocity, this study integrates morphological measurements with comparative genomics methods. Based on PacBio HiFi, ONT, and Hi-C technologies, chromosome-level T2T genomes of *H. bleekeri* and *H. leucisculus* were constructed, and comparative analyses were conducted on this basis. Through comparative genomic analysis, we systematically investigated the genetic basis underlying body size regulation and reproductive strategies in these two species. By integrating phenotypic and genotypic evidence, this study aims to elucidate the adaptive evolutionary patterns in response to distinct flow velocity.

## 2. Materials and Methods

### 2.1. Morphometrics

**Figure 1:**
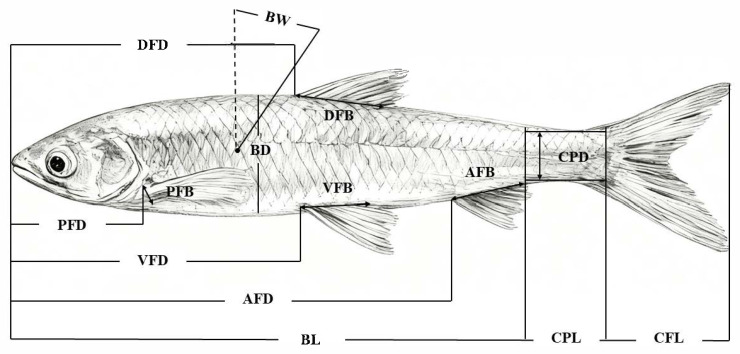
Morphometric parameters investigated for *H. bleekeri* and *H. leucisculus*. BL, Body length; BD, Body depth; BW, Body width; DFD, the tip of the lips to the starting point of the dorsal fin; PFD, the tip of the lips to the starting point of the pectoral fin; VFD, the tip of the lips to the starting point of the Ventral Fin; AFD, The tip of the lips to the starting point of the Anal Fin; DFB, Dorsal fin base length; PFB, Pectoral fin base length; VFB, Pelvic fin base length; AFB, Anal fin base length; CFL, Caudal fin length; CPL, Caudal peduncle length; CPD, Caudal peduncle depth.

In 2024 and 2025, specimens of *H. bleekeri* and *H. leucisculus* were collected from the Anqing, Yangzhou and Zhangjiagang sections of the Yangtze River, the Zhanghe River, and the Longwangshan Reservoir for morphological analysis. *H. bleekeri* was collected from lotic environments in the Anqing, Yangzhou, and Zhangjiagang sections of the Yangtze River. Based on long-term monitoring data from our early-resource surveys, the Yangtze mainstem maintains perennial flow, with average velocities ranging from 0.54 to 1.4 m/s in the Anqing section and reaching up to 1.81–3.04 m/s in the Taizhou section, representing moderate to high velocity habitats. In contrast, *H. leucisculus* was sampled from two sections: the Longwangshan Reservoir, a typical lentic environment where water remains nearly static under normal conditions (design discharge of 6.0 m^3^/s is reserved only for flood release), and the Zhanghe River, a major tributary with moderate current. This sampling design captures distinct flow regimes, providing a basis for comparing morphological adaptations to flow velocity. A total of 139 individuals were measured, including 78 *H. bleekeri* and 61 *H. leucisculus*. Specimens were identified with reference to the taxonomic descriptions provided by Ni et al. [10]. Fourteen morphometric characteristics were measured for each specimen (Figure 1). Morphometric data were recorded to an accuracy of 1 mm. Principal component analysis (PCA) was conducted to evaluate the 14 morphometric characteristics, which were corrected by standard body length to eliminate size-related effects [19]. A factor loading threshold of |±0.60| (≥36% explained variance) was set to screen variables with significant contributions to the extracted principal components [20]. All procedures involving animals were conducted following the guidelines for the Animal Care and Use Committee of the Freshwater Fisheries Research Center, Chinese Academy of Fishery Sciences. All animal welfare and sampling procedures were carried out in accordance with the Guiding Principles on Laboratory Animal—Guideline for Ethical Review of Animal Welfare (GB/T35892-2018) [21], issued by the Technical Committee for Laboratory Animal Sciences under the Standardization Administration of China (SAC/TC281).

### 2.2. Sample Collection for Genome Sequencing

**Figure 2:**
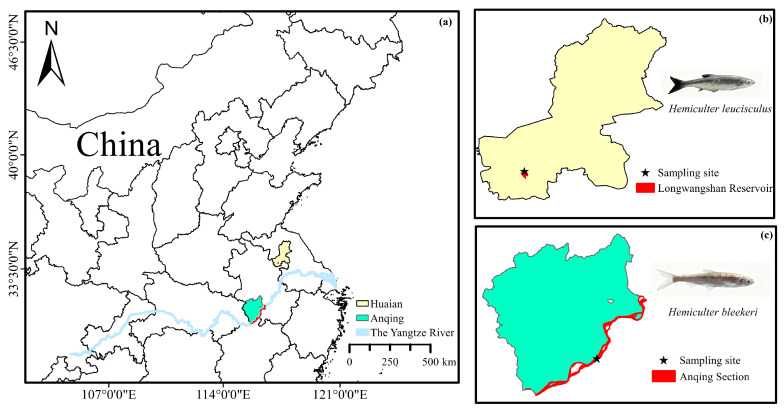
Sampling locations and specimen photographs of *H. bleekeri* and *H. leucisculus* used for genome sequencing. (**a**) In the maps, water bodies are shaded in red, and the sampling locations are marked with black stars. (**b**) *H. leucisculus*. (**c**) *H. bleekeri*.

In May 2024, a female adult *H. bleekeri* was collected for genome sequencing from the Anqing section of the Yangtze River (30.4927° N; 117.0023° E) in Anhui Province, China (Figure 2c). The sample collection was conducted under the project “Monitoring of Aquatic Resources in Key Waters of Anhui Province Project” (2023AHNYNC016XQ), approved by the Anhui Provincial Department of Agriculture and Rural Affairs, with the authorization from the Special Fishing License ([2024] No. 001). Additionally, the genome assembly was performed using a female individual of *H. leucisculus* collected from the Longwangshan Reservoir (32.9355° N; 118.5708° E) in Xuyi County, Jiangsu Province, China (Figure 2b). The body lengths of these two species are 107.11 mm and 147.33 mm, respectively, with corresponding body weights of 13.2 g and 43.7 g. Muscle tissue samples were taken from the region beneath the dorsal fin, were immediately frozen in liquid nitrogen, and stored at −80 °C prior to extracting genomic DNA.

### 2.3. Library Construction and Genome Sequencing

Genomic DNA was extracted using the standard phenol-chloroform method according to the manufacturer’s instructions. The quality and integrity of the isolated DNA were examined via agarose gel electrophoresis, while its concentration and purity were determined using a NanoDrop One spectrophotometer (Thermo Fisher Scientific, Waltham, MA, USA). Comprehensive whole-genome sequencing was carried out utilizing a combination of Nanopore ultra-long (Oxford Nanopore Technologies plc, Oxford, UK), PacBio HiFi(Paciffc Biosciences of California, Inc., Menlo Park, CA, USA), Hi-C (MGI Tech Co. Ltd., Shenzhen, China), and next-generation sequencing platforms. All library preparation and sequencing processes were carried out by OneMore Tech Ltd. (Wuhan, China).

High-quality genomic DNA was used to construct the sequencing libraries. For PacBio HiFi sequencing, two libraries with a 20 kb insert size were generated following the protocol of the SMRTbell Express Template Prep Kit 2.0 (PacBio Biosciences, CA, USA) and subsequently sequenced on a PacBio Sequel II platform. High-fidelity (HiFi) sequencing reads were produced using CCS software (v6.0.0) (https://github.com/pacificbiosciences/unanimity, accessed on 20 August 2024) under default settings. For the genome survey, a paired-end short-read library was prepared and sequenced on the DNBSEQ-T7 platform with 150 bp paired-end reads. The ultra-long ONT libraries were constructed using the Oxford Nanopore SQK-ULK001 kit (Oxford Nanopore Technologies plc, Oxford, UK). The raw reads were processed by filtering out low-quality sequences with mean quality scores below 7. Reads shorter than 10 kb were removed using Filtlong (v0.2.4, https://github.com/rrwick/Filtlong, accessed on 30 September 2024), and adaptor sequences were eliminated with Porechop v0.2.4 (https://github.com/rrwick/Porechop, accessed on 30 September 2024) [20]. Contig scaffolding was carried out using Hi-C technology. Genomic DNA was initially cross-linked with 1% formaldehyde for 20 min at 20–25 °C, followed by digestion with the Mbol restriction enzyme (400 U) and labeling with biotinylated nucleotides, then subjected to proximity ligation. Following reversal of the cross-links, the DNA was fragmented to 300–500 bp in size, and ligation junctions were enriched using streptavidin-coated beads. The Hi-C libraries were developed following the established Hi-C protocol and sequenced using the DNBSEQ-T7 platform, which was also employed for the paired-end BGI sequencing with short reads. The DNA concentration was quantified using a Qubit 4 fluorometer (Thermo Fisher Scientifc, Waltham, MA, USA), and sequencing of the Hi-C library was performed on the DNBSEQ-T7 platform.

Total RNA was isolated from various tissues, including muscle, liver, eye, brain, spleen, gonads, gills, heart and kidney, using TRIzol reagent in combination with the RNAprep Pure Tissue Kit (TIANGEN Biotech, Shanghai, China). Following evaluation of RNA integrity and purity, suitable RNA samples were selected for cDNA synthesis, fragmentation, and subsequent sequencing library preparation. Once the libraries met the required quality standards, they were subjected to high-throughput sequencing on the DNBSEQ-T7 platform.

### 2.4. T2T Genome Assembling and Quality Assessment

BGISEQ-T7 sequencing generated paired-end reads filtered with fastp (v0.23.2; ≥50 bp retained) with default settings [22]. The genome size and the heterozygosity of the genome were estimated with GenomeScope [23] on the basis of the 17-mer profile computed by Jellyfish2 [24]. The highly accurate long reads (approximately 15 kb in length, with accuracy exceeding 99%), including HiFi and Hi-C data, were assembled using Hifiasm (v0.19.6) to produce two primary genomes along with two draft contig-level assemblies [25]. Subsequently, quality-filtered short reads were aligned to the assembled genome using BWA-MEM under default parameters [26], and the genome sequence was refined through Pilon based on the resulting alignment data [27].

To assign contigs to chromosomes, low-quality reads and adapter sequences were initially filtered out using fastp (v0.23.2). Subsequently, the HiCUP pipeline was applied to generate a non-redundant contact matrix for chromosomal anchoring [28]. Subsequently, the contigs were anchored onto chromosomes using a 3D-DNA pipeline [29]. Manual scaffolding and orientation of the initially assembled chromosomes were performed using Juicebox Assembly Tools [30]. Subsequently, to produce a complete, gap-free genome assembly, ONT long reads were employed to close the remaining gaps in the Hi-C-assisted assembly using TGS-GapCloser (v1.2.0, https://github.com/BGI-Qingdao/TGS-GapCloser, accessed on 10 November 2024) [31]. Following this, Pilon was applied to correct potential errors in the extended, gap-filled genome using short-read sequencing data. Using the telomere database (http://telomerase.asu.edu/sequences_telomere.html, accessed on 15 December 2024), the repeat motifs 5′-CCCTAAA and 3′-TTTAGGG were identified within individual reads and subsequently reassembled to construct consensus sequences, enabling the generation of a telomere-to-telomere (T2T) complete genome assembly. Centromere regions were identified based on the distribution of tandem repeats using the multi-model prediction module in QuarTeT. The completeness of genome assembly was evaluated by Benchmarking Universal Single Copy Orthologs (BUSCO, v5.0.0) [32]. Genome continuity was assessed using the N50 values of the assembled contigs. Assembly accuracy was evaluated by calculating the mapping rate and genome coverage of both short reads and PacBio reads, aligned to the assembly using BWA-MEM under default parameters.

### 2.5. Repetitive Sequences Annotation, Gene Prediction and Functional Annotation

Repetitive elements of the *Hemiculter* genomes were identified by integrating homolog-based and de novo prediction strategies. First, tandem repeats were identified using Tandem Repeat Finder (v4.0.9) and long terminal repeats (LTRs) were identified using LTR Finder (v1.0.7) with default settings [33]. Repetitive elements were initially identified through the RepBase database (https://www.girinst.org/repbase/, accessed on 23 December 2024) using RepeatProteinMasker (v4.1.0) and RepeatMasker (v4.0.9) with default parameters to detect transposable element (TE)-associated protein repeat sequences. Subsequently, a de novo repeat library was constructed using RepeatModeler (v1.0.11) [34]. All predicted repetitive elements were integrated, and redundant sequences were eliminated to generate the final non-redundant set of repetitive elements.

Genes of *H. bleekeri* and *H. leucisculus* were predicted using following three methods: ab initio prediction, homology prediction and transcriptome sequencing. For ab initio prediction, Augustus v3.5.0 was used to predict ab initio genome models [35]. For homology prediction, the protein sequences of three species, *Chanodichthys erythropterus* (GCF_024489055.1), *Ctenopharyngodon idella* (GCF_019924925.1) and *Megalobrama amblycephala* (GCF_018812025.1) were downloaded from the National Center for Biotechnology Information (NCBI). Homologous protein sequences from related species were aligned to the newly assembled genome using BLAST (v2.2.28) [36] to infer gene structures. In parallel, transcriptome-based predictions were generated by mapping RNA-sequencing data to the reference genome with Hisat2 (v2.2.1) and Minimap2 (v2.26), followed by transcript reconstruction using Stringtie v2.2.1 [37,38]. All the predicted gene sets were integrated and verified to obtain the final dataset.

To systematically identify non-coding RNAs (ncRNAs) in the genome, a comprehensive approach combining multiple methods tailored to the structural and evolutionary characteristics of each RNA class was implemented. First, tRNAs were predicted across the entire genome using tRNAscan-SE (v 1.3.1) [39], which detects conserved tRNA secondary structure motifs. Next, owing to the high degree of evolutionary conservation exhibited by rRNA sequences, rRNA regions were identified through BLASTN alignments using rRNA sequences from closely related species as references. Finally, miRNA and snRNA genes were annotated by scanning the genome with covariance models sourced from the Rfam database (v14.8), employing the INFERNAL software suite. Collectively, these complementary approaches enabled a comprehensive annotation of major ncRNA elements within the genomes [40].

To acquire the functional annotation of the predicted gene sequences, we employed multiple databases for comprehensive annotation. These databases included the NCBI non-redundant protein database (NR), InterPro, Swiss-Prot, TrEMBL, the Clusters of Orthologous Groups of proteins in eukaryotes (KOG), the Kyoto Encyclopedia of Genes and Genomes (KEGG), and the Protein Family Database (Pfam). Protein domain analysis was performed using InterProScan (v5.61-93.0) [41]. Additionally, DIAMOND v2.0.14 [42] software was utilized to compare gene sequences against the KEGG, Swiss-Prot, TrEMBL, KOG, and NR databases with cutoff e-values of 1 × 10^−5^.

### 2.6. Phylogenetic Analysis, Estimation of Divergence Time and Collinearity Analysis

For clustering gene families, 16 species, including *H. bleekeri, H. leucisculus, Danio rerio*, *Culter alburnus*, *Megalobrama amblycephala*, *Cyprinus carpio* and *Oryzias latipes*, were selected for gene family analysis (Supplementary Table S1). Orthofinder v2.3.12 [43] was employed to perform gene family clustering across 16 species, enabling the identification of orthologous, paralogous, and single-copy homologous genes. The OrthoMCL v2.0.9 [44] software was used to the orthologous protein families with default settings based on the filtered (e-value < 1 × 10^−5^) all-vs-all blastp results. The construction of the species phylogenetic tree was performed using RAxML v8.2.12 [45] software under the maximum-likelihood framework (“-f a -N 100 -m GTRGAMMA”) with 1000 bootstrap replicates. Based on the calibration times from the TimeTree database, the MCMCtree in the software PAML package (v4.9) [46] was used to estimate the divergence times for the species. Fossil-based calibration constraints included the divergence between *Takifugu rubripes* and *Oryzias latipes* (103.8–130.8 Mya), *Danio rerio* and *Zacco platypus* (42.3–68.7 Mya), and *Parabramis pekinensis* and *Culter alburnus* (4.1–8.0 Mya) [47].

The WGDI v0.5.6 [48] software was used to perform collinearity analysis on the genomes of *H. bleekeri* and *H. leucisculus*. An all-against-all BLASTP v2.11.0 search was performed on the filtered dataset with an E-value threshold of 1 × 10^−5^ to detect sequence similarities among proteins. The genomic positions of homologous gene pairs were then identified using JCVI software v1.1.22 [49] and all genes located within syntenic blocks were extracted.

### 2.7. Gene Family Contraction and Expansion

Based on the species phylogenetic tree and gene family clustering results, the birth-death model was applied to infer the ancestral gene family sizes across branches, and gene family expansions and contractions relative to the inferred ancestors were predicted. CAFE (v5.0.0) [50] software with parameters set to “-P 0.05 -E 2 -I 300 -R 1” was used to identify expanded and contracted gene families in the species. Significant expansion or contraction was defined at *p* < 0.05, and KEGG pathway and Gene Ontology (GO) enrichment analyses were conducted for the genes within these families.

## 3. Results

### 3.1. Morphometric Measurements

To elucidate the overall morphometric distinctions between *H. bleekeri* and *H. leucisculus*, we employed both principal component analysis (PCA) and *t*-tests to compare 14 body-length-standardized morphometric variables. The results revealed that *H. bleekeri* exhibited a significantly lower BD/BL ratio (0.205 ± 0.014) than *H. leucisculus* (0.219 ± 0.024), indicating a shallower body depth. Similarly, the DFD/BL ratio was significantly smaller in *H. bleekeri* (0.494 ± 0.018) than in *H. leucisculus* (0.555 ± 0.068), reflecting a more anterior insertion of the dorsal fin (Table 1). This morphometric configuration suggests a more slender and streamlined body shape in *H. bleekeri*. From a hydrodynamic perspective, body depth (BD) is closely linked to swimming performance; according to the principles of fluid dynamics, fish inhabiting high-flow velocity typically evolve slender body forms to minimize drag during both steady swimming and rapid acceleration when attacking prey [51,52]. The posteriorly positioned dorsal fin in *H. leucisculus*, in contrast, is a trait associated with enhanced stability in lentic environments, as it contributes to stabilization and braking during acceleration [53]. In summary, the divergent morphologies in body depth and dorsal fin placement between these two species reflect distinct ecological specializations that correspond to their respective hydrodynamic environments.

### 3.2. Genome Sequencing and T2T Gap-Free Assembly

**Figure 3:**
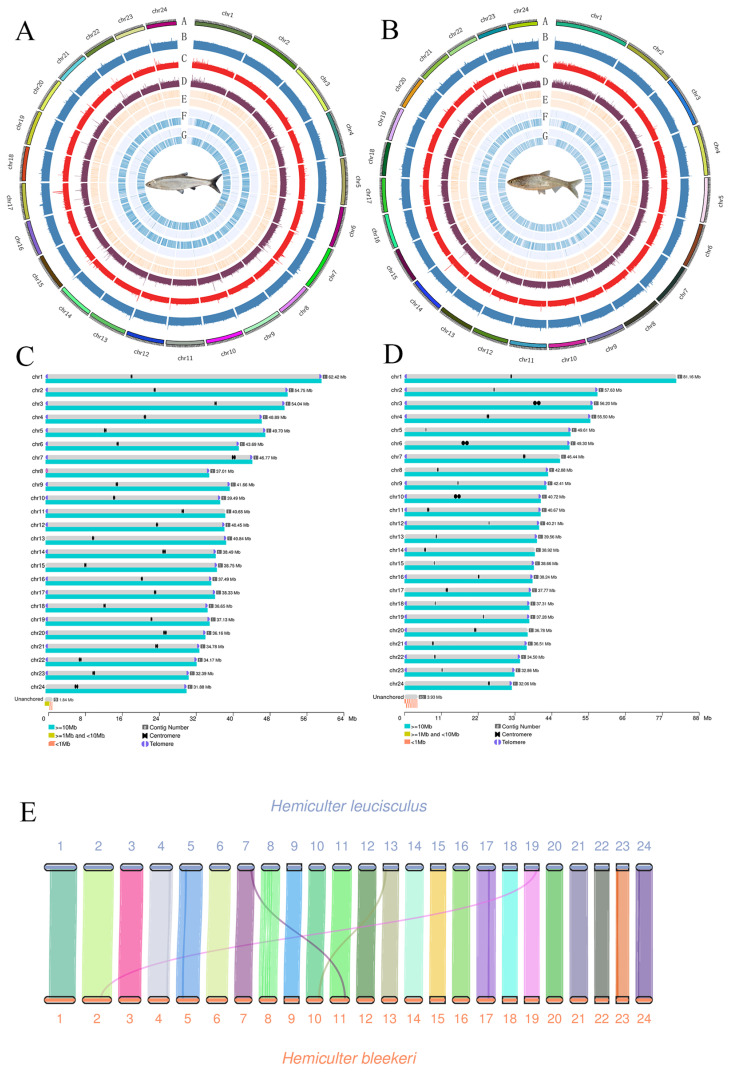
The genome assemblies and synteny of *H. bleekeri* and *H. leucisculus*. (**A**,**B**) Circular genome diagrams of *H. bleekeri* (**left**) and *H. leucisculus* (**right**). (**C**,**D**) Telomere-to-telomere (T2T) assembly representation of *H. bleekeri* (**left**) and *H. leucisculus* (**right**). (**E**) Genome collinearity analysis between the two species.

We integrated PacBio HiFi long reads, ultra-long ONT reads and Hi-C sequencing data to generate chromosome-level genome assemblies for *H. bleekeri* and *H. leucisculus*. K-mer analysis estimated the genome sizes of *H. bleekeri* and *H. leucisculus* to be approximately 953 Mb and 981 Mb, respectively (Supplementary Table S2, Supplementary Figure S1). For *H. bleekeri*, we generated approximately 51.49 Gb (51×) PacBio HiFi reads, 106.57 Gb (106×) Hi-C reads and 79.61 Gb (79×) ONT reads. For *H. leucisculus*, we generated 50.83 Gb (48×) of PacBio HiFi reads and 105.31 Gb (100×) Hi-C reads, supplemented by an additional 45.77 Gb (43×) of ONT reads. (Supplementary Table S3). Assessment of the Hi-C interaction maps confirmed high-quality genome assemblies for both species. We observed dense, distinct diagonal contours, representing strong intra-chromosomal contacts, and a clear background with scant inter-chromosomal noise (Supplementary Figure S2). These hallmarks of a robust Hi-C map provide strong support for the correct ordering and orientation of scaffolds at the chromosome level [54] (Supplementary Tables S4 and S5). The *H. bleekeri* genome assembly comprised 24 scaffolds, with both contig N50 and scaffold N50 of 40.45 Mb. The final genome assembly was 0.998 Gb, with a heterozygosity rate of 1.51%. Similarly, the *H. leucisculus* genome assembly consisted of 24 scaffolds, with both contig N50 and scaffold N50 measuring 40.66 Mb. These scaffolds were also assembled into a 1.05 Gb genome, with a heterozygosity rate of 1.10% (Table 2). Using a four-base telomeric repeat sequence as a query, we identified 48 telomeres were annotated across 24 chromosomes in *H. bleekeri*, while 37 telomeres were annotated across 24 chromosomes in *H. leucisculus*. Telomeric repeat units of the *H. bleekeri* genome were detected at both ends of 24 chromosomes. Similarly, telomeric repeat units of the *H. leucisculus* genome were detected at both ends of 13 chromosomes and at 1 end of 11 chromosomes (Supplementary Tables S6 and S7). Finally, we further employed Hi-C data to facilitate chromosome-level assembly and orientation, ultimately generating a complete Hi-C interaction matrix that clearly reflects large-scale genomic interactions across the entire genome (Figure 3). Centromere positions were identified using quarTeT-based predictions, Hi-C contact signal patterns, and element density profiles. All of the chromosomes were assembled as gap-free chromosomes. The positions of the identified telomeres on the chromosomes and their distribution across the contigs were annotated and displayed. Thus, the *H. bleekeri* and *H. leucisculus* genomes can be considered high-quality telomere-to-telomere (T2T) assemblies.

Multiple data types and methods were implemented to evaluate the quality of the *H. bleekeri* and *H. leucisculus* genome assembly. The interaction matrix generated from the Hi-C short-read library indicated that all 24 chromosomes were fully and reasonably assembled (Figure 3). BUSCO evaluation showed that both species had a high percentage (99.4%) of complete BUSCOs. The assembly consensus quality value (QV) was 43.15 for *H. bleekeri* and 44.39 for *H. leucisculus*, demonstrating high integrity and accuracy of both genomes.

### 3.3. Gene Prediction and Annotation

Three strategies, namely the ab initio prediction method, the homology prediction method and the prediction based on transcriptome data, were applied to annotate repeat elements. The genomes of *H. bleekeri* and *H. leucisculus* contained 567.82 Mb (54.23%) and 535.56 Mb (53.63%) of repetitive sequences, respectively (Supplementary Table S8). Repetitive sequence annotation demonstrated transposable elements (TEs) constituted 51.78% (516.93 Mb) of the *H. bleekeri* genome and 52.19% (546.45 Mb) of the *H. leucisculus* genome (Supplementary Table S9). In total, 26,168 genes were identified in *H. bleekeri*, and 26,446 genes were identified in *H. leucisculus* (Table S2). A total of 25,914 (99.27%) and 26,300 (99.45%) gene models in *H. bleekeri* and *H. leucisculus*, respectively. We successfully annotated at least one of the major databases (NR, SwissProt, KOG, TrEMBL, TF, Pfam, InterPro, GO, or KEGG), providing a robust foundation for subsequent functional analyses [55] (Supplementary Table S10). We identified four types of non-coding RNAs in the genome of *H. bleekeri*: 2608 miRNAs, 18,593 tRNAs, 10,276 rRNAs and 1859 snRNAs. In the genome of *H. leucisculus*, we identified four types of non-coding RNAs: 690 miRNAs, 14,094 tRNAs, 12,179 rRNAs and 1473 snRNAs (Supplementary Tables S11 and S12). In conclusion, our annotation of two gene sets of reliably high quality have established a robust foundation for further research.

### 3.4. Collinearity Analysis of H. bleekeri and H. leucisculus

Collinearity analysis of the *H. bleekeri* and *H. leucisculus* genomes was conducted based on the annotation file. Intraspecific self-comparison analysis revealed the presence of 726 and 844 syntenic blocks in the genomes of *H. bleekeri* and *H. leucisculus*, respectively, with an average of eight gene pairs identified per block. The total number of gene pairs amounted to 5709 in *H. bleekeri* and 6552 in *H. leucisculus*, suggesting a substantial level of structural conservation within the genomes of both fish species (Supplementary Table S13). The median block lengths of both species are comparable, with values ranging from 370,213 to 384,466 bp in *H. bleekeri* and from 355,077 to 358,658 bp in *H. leucisculus*. The results indicate that *H. bleekeri* and *H. leucisculus* exhibit extremely high collinearity.

### 3.5. Gene Family Clustering and Phylogenetic Analysis

**Figure 4:**
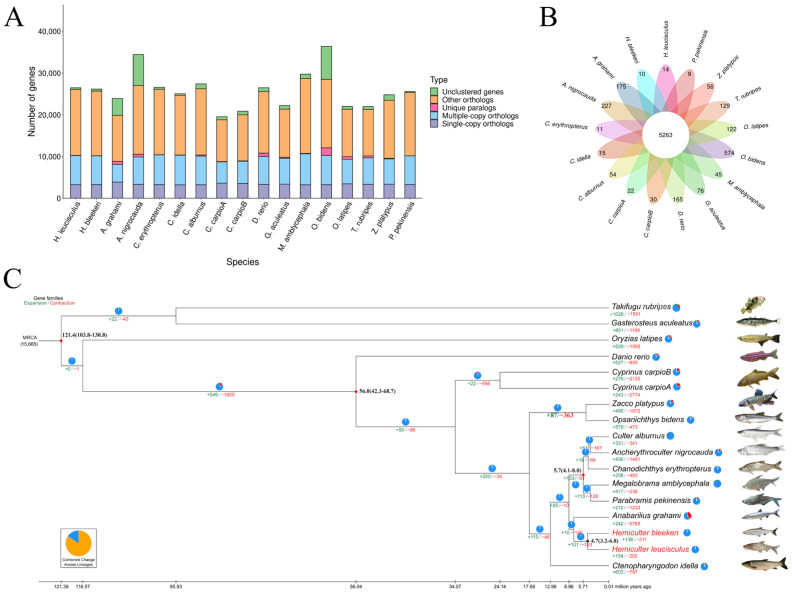
Phylogeny and gene family analysis of 16 fish species. (**A**) Number of homologous genes shared among the species. (**B**) Venn diagram of gene family clustering, with numbers indicating the count of gene families. (**C**) A phylogenetic tree of 16 fish species. Estimated divergence times (in million years ago, Mya) are shown in black at each node. Pie charts and associated colored values represent the proportion and absolute number of expanded (+, green) and contracted (−, red) gene families, respectively. Green numerals along the branches indicate the number of expanded gene families, while red numerals denote the number of contracted gene families.

Orthologous gene families, including both single-copy and multi-copy families, were defined based on the predicted proteomes of *H. bleekeri* and *H. leucisculus* from this study, together with those of 14 other fish species, namely *Danio rerio*, *Culter alburnus*, *Megalobrama amblycephala*, *Cyprinus carpio* and *Oryzias latipes*, etc. (Figure 4 and Supplementary Table S1). A total of 2440 single-copy genes were identified and subsequently used for phylogenetic tree construction (Supplementary Table S14). Moreover, the divergence time analysis showed that *H. bleekeri* and *H. leucisculus* diverged from the ancestor of *A. grahami* approximately 7.8 million years ago (Mya). The divergence between *H. bleekeri* and *H. leucisculus* was estimated at approximately 4.7 (3.2–6.8) Mya (95% confidence interval [CI]: 3.2–6.8 Mya, Figure 4C). These findings provide insights into the evolutionary history and genetic relationships within the *Hemiculter*.

### 3.6. Comparative Genomic Analysis Underlying Morphological Divergence

Our comparative genomic analysis further revealed significant gene family expansions in *H. bleekeri* involving key genes related to skeletal development and body shape determination, namely *b3glct* (beta-1,3-glucosyltransferase), *tgm* (Protein-glutamine gamma-glutamyltransferase), *jarid* (protein Jumonji), and *ppara* (Peroxisome proliferator-activated receptor). These findings provide a compelling genetic basis for the slender and streamlined body form identified through our morphometric analyses. The *b3glct* gene encodes an enzyme critical for the synthesis of extracellular matrix components. Its expansion may alter the properties of connective tissues, including cartilage and bone, potentially facilitating a more elongated and flexible body framework [56,57]. The *tgm* gene family encodes transglutaminases, which are enzymes involved in cross-linking proteins in the extracellular matrix. This process is essential for bone mineralization and stability. The expansion of *tgm* could lead to modifications in skeletal structure that contribute to a leaner body plan [58]. The *jarid* gene, a histone demethylase, acts as a key epigenetic regulator of development. It influences the expression of downstream genes controlling axial patterning and somite formation—fundamental processes that determine the overall body shape and the number and size of vertebrae [59]. The *ppara* gene is a master regulator of lipid and energy metabolism. While not directly a structural gene, its expansion might influence body depth by modulating fat deposition and distribution along the body axis, thereby promoting a more streamlined profile [60].

Critically, the coordinated expansion of these specific genes is not arbitrary. A genome-wide association study (GWAS) on the body length -to-body depth (BL/BD) ratio in the large yellow croaker independently identified *b3glct*, *tgm*, *jarid*, and *ppara* as candidate genes, highlighting their evolutionarily conserved role in shaping fish body morphology [61]. We propose that in *H. bleekeri*, the expansion of this suite of genes has been subject to natural selection to establish a skeletal and metabolic framework that is inherently more slender. This genetic predisposition directly translates into the reduced body depth (BD/BL) and anteriorly positioned dorsal fin observed in morphometric analyses [62].

This slender morphology represents a classic adaptation to high-flow velocity. From a hydrodynamic perspective, a streamlined body minimizes drag and pressure resistance, enabling *H. bleekeri* to maintain position and maneuver efficiently in flow velocity environments with less energy expenditure. Therefore, the genomic signatures we uncovered do not merely correlate with but are likely the evolutionary drivers behind the specialized body form that enhances fitness in its specific lotic habitat. Integrative evidence from morphology and genomics strongly indicates that the genetic regulatory mechanisms underlying skeletal development are among the core drivers shaping its ecologically adaptive phenotypes.

### 3.7. Comparative Genomic Analysis Underlying Reproductive Strategies

**Figure 5:**
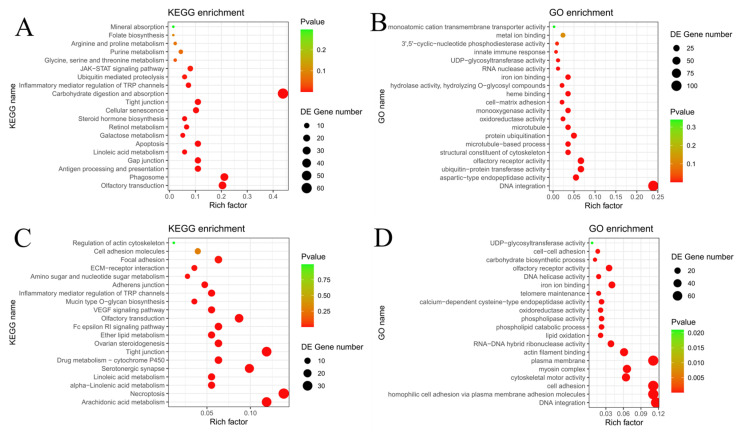
Functional enrichment of genes. (Dot size represents the number of enriched DEGs). (**A**) Enriched KEGG Pathway of Expanded Genes in *H. bleekeri*. (**B**) Enriched GO Pathway of Expanded Genes in *H. bleekeri.* (**C**) Enriched KEGG Pathway of Expanded Genes in *H. leucisculus.* (**D**) Enriched GO Pathway of Expanded Genes in *H. leucisculus*.

Gene families that have undergone expansion may develop novel functions and contribute to the development of new metabolic pathways, thereby enhancing the ability of fish to adapt to varying water flow velocity in their habitats. To reveal the genetic basis of fish adaptation to different flow velocity, we conducted an analysis of gene family evolution based on orthologous clusters of protein-coding sequences from 16 species. In *H. bleekeri*, a total of 136 expanded and 211 contracted gene families were identified, among which 80 (encompassing 576 genes) and 70 (encompassing 92 genes) were identified as significantly expanded and significantly contracted gene families, respectively. Based on these findings, functional enrichment analyses were subsequently performed using the GO and KEGG databases to gain deeper insights into the biological roles and pathways associated with these gene families. The expanded gene families were enriched in GO terms and KEGG pathways, including terms such as Ubiquitin mediated proteolysis (ko04120), Mineral absorption (ko04978), Galactose metabolism (ko00052), JAK-STAT signaling pathway (ko04630) (Figure 5A). Furthermore, corroborating evidence comes from expanded Gene Ontology (GO) terms, including ubiquitin-protein transferase activity (GO:0004842), aspartic-type endopeptidase activity (GO:0004190), and metal ion binding (GO:0046872) (Figure 5B). GO and KEGG enrichment analyses indicated that the expanded gene families associated with reproduction in *H. bleekeri* constitute a crucial molecular basis for its adaptation to flow velocity and for the production of pelagic eggs [63].

A total of 154 expanded and 202 contracted gene families were identified in *H. leucisculus*. Of these, 92 expanded families (888 genes) and 70 contracted families (52 genes) exhibited significant changes. Enrichment analysis of KEGG pathways and GO terms associated with reproduction in *H. leucisculus* revealed expansions in gene families related to cell adhesion, expansions were identified in KEGG pathways including Adherens junction (ko04520), Amino sugar and nucleotide sugar metabolism (ko00520), Mucin type O-glycan biosynthesis (ko00512) and Regulation of actin cytoskeleton (ko04810) (Figure 5C). These findings were strongly supported by concurrent expansions in GO terms such as homophilic cell adhesion via plasma membrane adhesion molecules (GO:0007156), actin filament binding (GO:0051015) and carbohydrate biosynthetic process (GO:0016051) (Figure 5D). The analyses provide key molecular evidence supporting the successful adhesion of its eggs and the stability of its stable reproductive strategy in flowing water environments [64,65].

## 4. Discussion

### 4.1. Genome Assembly Quality

Long-read sequencing enables the generation of continuous DNA sequences exceeding 10 kb in length, offering a powerful approach for genomic investigation and facilitating the achievement of gap-free genome assembly [66]. Such complete genomes allow comprehensive characterization of genomic content and are widely considered the gold standard in genome assembly. In contrast to Second-generation sequencing-based technologies, third-generation Sequencing can effectively traverse highly repetitive genomic regions and resolve assembly gaps that remain intractable with short-read methods, thereby substantially enhancing genome contiguity [67]. In this study, we selected *H. bleekeri* and *H. leucisculus* as representative species and integrated multiple advanced sequencing technologies, including PacBio HiFi long reads, ultra-long ONT reads and Hi-C sequencing data, into a hybrid assembly framework to generate high-quality, gap-free genome assemblies for both species. The final assembled genomes were 0.998 Gb and 1.05 Gb for *H. bleekeri* and *H. leucisculus*, respectively, each comprising 24 chromosomes represented as single contigs without gaps, meeting the stringent criteria for gap-free assembly. Genome completeness was assessed via BUSCO, yielding scores of 99.34% for both species, confirming exceptional genomic integrity. Concomitant with ongoing advancements in PacBio and Nanopore sequencing technologies, key assembly metrics such as contig N50 have markedly improved, progressing from several megabases (Mb) to over 100 Mb in recent high-quality assemblies. In this work, the contig N50 values reached 40.45 Mb for *H. bleekeri* and 40.66 Mb for *H. leucisculus*, comparable to those reported for other members of the Culterinae‌, including *Culter alburnus* (N50: 32.9 Mb) [68] and *Chanodichthys mongolicus* (female N50: 34.4 Mb; male N50: 34.6 Mb) [69], thus underscoring the high quality and reliability of the generated assemblies.

### 4.2. Genome Annotation Characteristics

Consistent with most fish genomes, the genomes of *H. bleekeri* and *H. leucisculus* exhibit a high proportion of repetitive elements. Genome-wide annotation of repetitive sequences revealed that these repeats are predominantly composed of four major classes: short interspersed nuclear elements (SINEs), long interspersed nuclear elements (LINEs), long terminal repeat (LTR) retrotransposons, and DNA transposons. Through an integrated approach combining multiple annotation methods to minimize redundancy, the total lengths of annotated repetitive sequences were determined to be 541.40 Mb and 567.82 Mb, respectively, accounting for 54.23% of each genome. This finding highlights the complex architecture of the repetitive content in both species. Protein-coding genes were annotated using a comprehensive strategy that combined homology-based prediction, de novo assembly, and RNA-Seq-supported evidence, yielding 26,146 and 26,446 genes for *H. bleekeri* and *H. leucisculus*, respectively. Comparative analysis with other teleost genomes showed that these gene counts are similar to those of *Culter alburnus* (26,208 genes) 67, a closely related species within the Culterinae, but lower than that of *Chanodichthys erythropterus* (33,706 genes) [70]. Functional annotation assigned putative functions to 25,955 and 26,300 protein-coding genes, representing 99.27% and 99.45% of the total gene sets, respectively, thereby providing a robust foundation for downstream functional genomics studies and pathway analyses. Collectively, these results demonstrate that the genome annotations generated in this study are of high quality. They represent valuable genomic resources for elucidating the genomic features and molecular mechanisms underlying the biology of *H. bleekeri* and *H. leucisculus*.

### 4.3. Comparative Genomics Provides Insights into Adaptation to Flow Velocity

Comparative genomic analysis has insights into the genetic basis underlying adaptive divergence in morphology and reproductive strategies between *H. bleekeri* and *H. leucisculus*. Morphometric, *H. bleekeri* exhibits significant expansions in multiple genes associated with body shape, including *b3glct*, *tgm*, *jarid* and *ppara*, which likely served as key drivers in the evolution of its streamlined body plan. These genes have been experimentally implicated in the regulation of BD/BL in species such as the large yellow croaker (*Larimichthys crocea*) [61], supporting their conserved role in fish body shape determination. This conservation implies that the observed gene family expansions in *H. bleekeri* may represent an adaptive evolutionary response shaped by natural selection to enhance fitness in flow aquatic environments. The streamlined body morphology reduces hydrodynamic resistance [67], enabling *H. bleekeri* to maintain station and execute efficient locomotion in flowing water with minimal energy expenditure.

With respect to reproductive strategies, the two fish species display distinct genetic adaptations, further elucidating the divergent evolutionary mechanisms by which *H. bleekeri* and *H. leucisculus* adapt to flow environments. In *H. bleekeri*, 136 expanded gene families were expanded and significantly enriched in several biological pathways and molecular functions. These included ubiquitin-mediated proteolysis, mineral absorption, ubiquitin transferase activity and metal ion binding [62]. These enriched pathways and functions facilitate maturation and buoyancy regulation of pelagic eggs, providing a molecular basis for their successful development and dispersal in flowing water. In contrast, *H. leucisculus* has 92 expanded gene families that are predominantly enriched in pathways related to actin cytoskeleton organization, as well as in functions such as homophilic cell adhesion and actin filament binding. These molecular features strongly correlate with the adhesive properties of its eggs [62]. Together, these findings highlight how gene family expansions underlie specialized reproductive strategies in response to shared environmental pressures.

### 4.4. Potential Factors Beyond Flow Velocity

We acknowledge that although this research emphasizes flow velocity as a major selective agent, adaptive differentiation in natural populations is typically shaped by a variety of ecological and evolutionary factors. For instance: (1) Habitat structure and substrate: rocky or coarse substrates common in high-flow habitats may promote the evolution of morphological features related to locomotion, such as body flexibility and muscle attachment structures. (2) Dietary differences: variation in prey composition across flow environments may drive divergence in feeding-related skeletal structures (e.g., jaws and pharyngeal bones), involving genes that regulate skeletal development and morphology. (3) Physicochemical water characteristics: differences in parameters such as water temperature, turbidity, and dissolved oxygen may further influence physiological adaptation and phenotypic shaping [71]. Thus, although flow velocity serves as the primary explanatory factor for the morphological differences observed here, the evolutionary trajectories of *H. bleekeri* and *H. leucisculus* have likely been shaped by the integrated influence of the above factors. Future studies could employ approaches such as whole-genome resequencing and tissue-specific expression profiles to systematically dissect the relative contributions of various environmental factors to species divergence.

## 5. Conclusions

This study suggests that flow velocity is a potential key environmental driver of adaptive differentiation in congeneric fish species. By examining two closely related species, *H*. *bleekeri* and *H*. *leucisculus*, which have adapted to distinct flow velocity. We systematically elucidated the molecular mechanisms underlying the influence of aquatic habitats on fish morphology and reproductive strategies through genotype phenotype association analyses. A major advancement of this study is the construction of high-quality, chromosome-level, T2T genome assemblies for both species, providing a robust genomic foundation for comparative genomic investigations. By integrating morphometric and genomic data, we identified adaptive traits that exhibit divergence between the two species and are associated with the hydrodynamic conditions of their respective habitats.

Through comparative genomic analysis of *H. bleekeri*, which is adapted to fast flow velocity environments, we identified genes associated with a streamlined body morphology as well as conserved pathways including ubiquitination and ion transport. These pathways are potentially linked to its reproductive strategy of producing pelagic eggs and collectively represent a candidate molecular mechanism underlying its functional adaptation to lotic habitats. In contrast, comparative genomic analysis of *H*. *leucisculus*, reveals enrichment in the glycosaminoglycan biosynthesis pathway. This finding suggests that the pathway may play a functional role in the development of adhesive eggs, consistent with its reproductive strategy of laying adhesive eggs in stable aquatic habitats. The gene family expansions identified in this study provide important genetic insights and indirect evidence for understanding the mechanisms underlying species adaptation; however, definitive confirmation of their functional roles will require targeted experimental validation in future studies. Future studies could employ gene editing techniques to functionally validate key genes, combined with population genomics to trace the origin and evolutionary dynamics of these adaptive traits.

Collectively, this study integrates high-quality chromosome-level genome sequencing with morphometric analysis to provide detailed insights into the adaptive divergence in body form and reproductive strategies between *H. bleekeri* and *H. leucisculus*. These findings provide potential genomic evidence for how flow velocity shapes morphological and reproductive adaptations in freshwater fishes.

## Figures and Tables

**Table 1:** Morphometric measurements of *H. bleekeri* and *H. leucisculus*.

Variables	Mean		PCA			*p*
	*H. bleekeri*	*H. leucisculus*	Principal Component 1	Principal Component 2	Principal Component 3	
BD/BL *	0.205 ± 0.014	0.219 ± 0.024	0.172	0.190	0.722	<0.01 **
BW/BL	0.096 ± 0.009	0.100 ± 0.025	0.203	−0.041	0.885	0.199
DFD/BL *	0.494 ± 0.018	0.555 ± 0.068	−0.156	0.975	−0.056	<0.01 **
PFD/BL	0.206 ± 0.028	0.225 ± 0.010	−0.044	0.382	0.067	<0.01 **
VFD/BL	0.460 ± 0.017	0.467 ± 0.422	0.094	0.270	−0.209	0.224
AFD/BL	0.701 ± 0.018	0.696 ± 0.028	0.174	−0.017	0.540	0.198
DFB/BL	0.103 ± 0.0133	0.093 ± 0.020	0.290	−0.387	0.514	<0.01 **
PFB/BL	0.043 ± 0.005	0.045 ± 0.006	0.074	0.194	0.386	0.004 **
VFB/BL	0.045 ± 0.006	0.038 ± 0.001	0.109	−0.391	0.170	<0.01 **
AFB/BL	0.158 ± 0.011	0.162 ± 0.043	−0.009	0.191	−0.735	0.336
CFL/BL	0.233 ± 0.126	0.200 ± 0.050	−0.023	−0.318	−0.853	<0.01 **
CPD/BL	0.093 ± 0.001	0.085 ± 0.008	0.434	−0.366	0.339	<0.01 **
CPL/BL	0.153 ± 0.015	0.147 ± 0.020	−0.703	−0.318	0.259	0.041 *
CPD/CPL	0.614 ± 0.071	0.592 ± 0.115	0.998	0.049	−0.024	0.152
Explained variability (%)	/	/	50.499	19.492	13.225	/
Accumulative variability (%)	/	/	50.499	69.991	83.216	/

* *p* < 0.05, ** *p* < 0.01. Abbreviations for morphometric characteristics are provided in Figure 1; A factor loading threshold of |±0.60| (corresponding to ≥36% explained variance) was applied to identify variables with substantial contributions to the PCs; Sample size (*n*) = 139.

**Table 2:** Genome assembly statistics of *H. bleekeri* and *H. leucisculus*.

Library Type	*H. bleekeri*	*H. leucisculus*
Total size of assembled genome (Gb)	0.998	1.05
Contig N50 (Mb)	40.45	40.66
Contig N90 (Mb)	34.17	36.51
Number of contigs	24	24
Scaffold N50 (Mb)	40.45	40.66
Scaffold N90 (Mb)	34.17	36.51
Scaffolds number	24	24
Number of base chromosomes	24	24
Number of gap-free chromosomes	24	24
Number of candidate telomeres	48	37
Number of gaps	0	0
Number of telomeres (pairs/single)	24/0	13/11
Genome BUSCOs	99.4%	99.4%
QV	43.15	44.39
TE size	50.66%	52.19%
GC content	37.82	37.42
Number of genes	26,168	26,446
Gene BUSCOs	99.4%	99.4%
Heterozygosity	1.51	1.10

## Data Availability

The sequencing dataset and genome assembly of *Hemiculter bleekeri* have been deposited in the NCBI SRA database under project number PRJNA1297333 (https://www.ncbi.nlm.nih.gov/bioproject/PRJNA1297333/ (accessed on 27 July 2025)). The sequencing dataset and genome assembly of *Hemiculter leucisculus* have been deposited in the ENA database under project number ERP185770 (https://www.ebi.ac.uk/ena/submit/webin/report/runs/ERP185770 (accessed on 28 November 2025)).

## Supplementary Materials

The following supporting information can be downloaded at: https://www.mdpi.com/article/10.3390/biom16010083/s1, Figure S1: K-mer distribution and assembly completeness assessment for *H. bleekeri* and *H. leucisculus*; Figure S2: Hi-C interaction maps for *H. bleekeri* and *H. leucisculus*; Table S1: Species information used in comparative genomic analyses; Table S2: Genome survey statistics for *H. bleekeri* and *H. leucisculus* based on K-mer analysis; Table S3: Summary Statistics of Sequencing Data for *H. bleekeri* and *H. leucisculus* Genome Assembly and Annotation; Table S4: The Hi-C assisted assembly for *H. bleekeri*; Table S5: The Hi-C assisted assembly of *H. leucisculus*; Table S6: The details of chromosome and number of telomere characteristic sequences for *H. bleekeri* genome; Table S7: The details of chromosome and number of telomere characteristic sequences for *H. leucisculus* genome; Table S8: Repetitive sequence statistics results of *H. bleekeri* and *H. leucisculus*; Table S9: Statistical results of the classification of repetitive sequences of *H. bleekeri* and *H. leucisculus*; Table S10: Prediction results of gene function of *H. bleekeri* and *H. leucisculus*; Table S11: Statistics result of Non-coding RNA annotation for *H. bleekeri*; Table S12: Statistics result of Non-coding RNA annotation for *H. leucisculus*; Table S13: Statistical analysis of collinearity results between *H. bleekeri* and *H. leucisculus*; Table S14: Statistics of gene family clustering results.
