# Supplementary figures and images for: Telomere-to-Telomere Genome Assembly of Two Hemiculter Species Provide Insights into the Genomic and Morphometric Bases of Adaptation to Flow Velocity
Source: Biomolecules. 2026 Jan 4;16(1):83. doi: 10.3390/biom16010083 (PMC12838669; doi:10.3390/biom16010083)

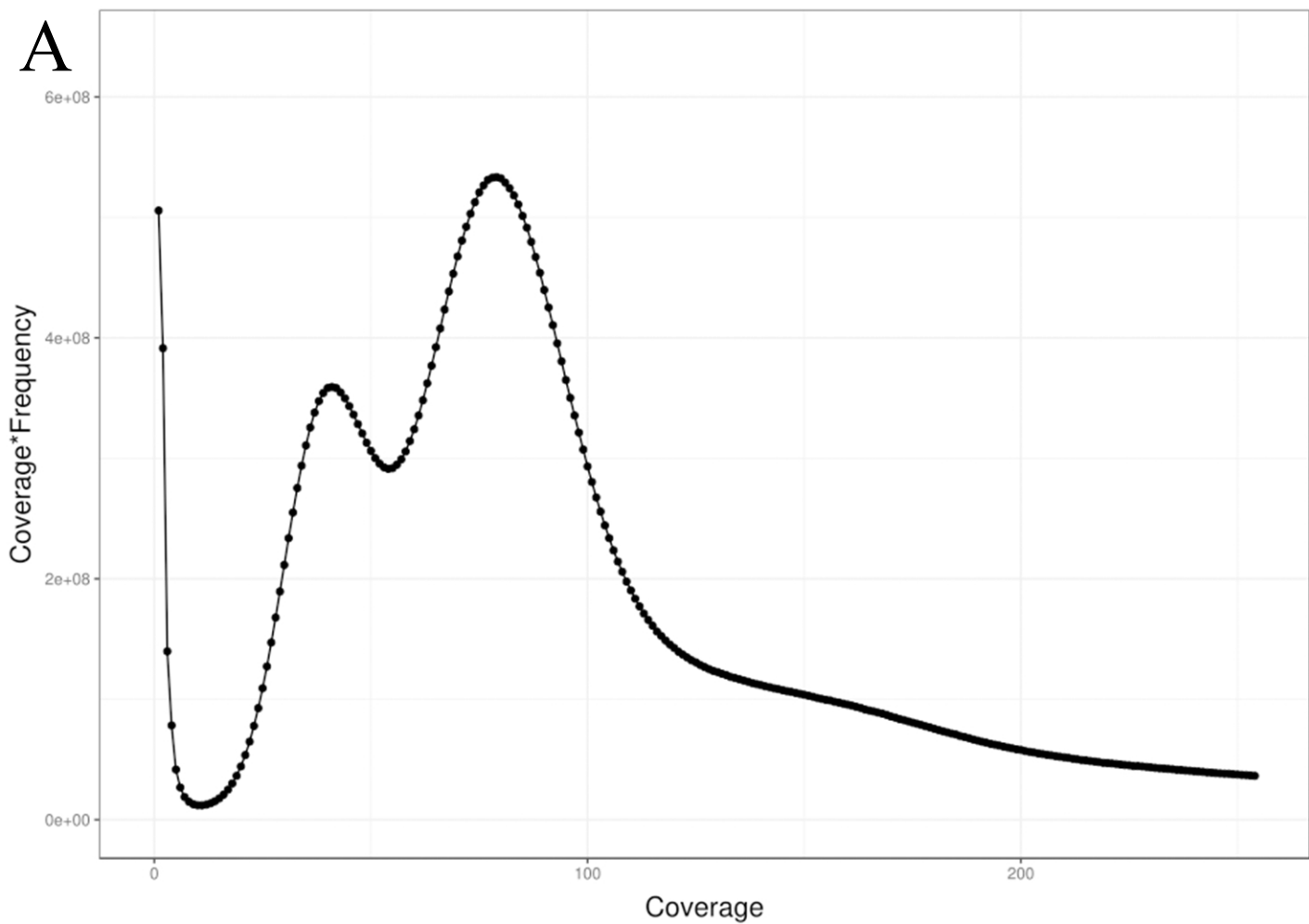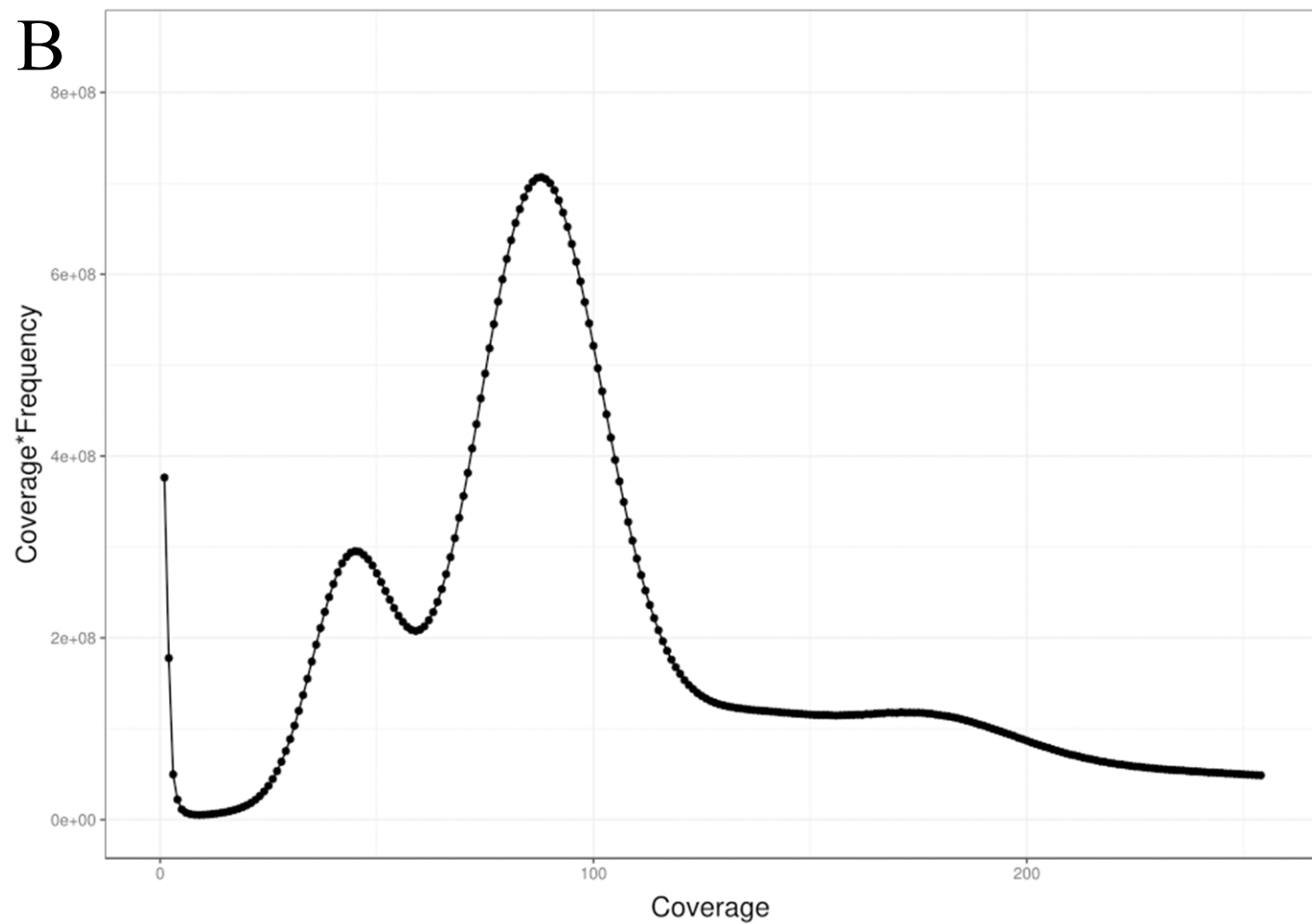

Supplement: Supplementary file 1 [file biomolecules-16-00083-s001.zip › Supplementary Files/Supplementary Figure 1.pdf]

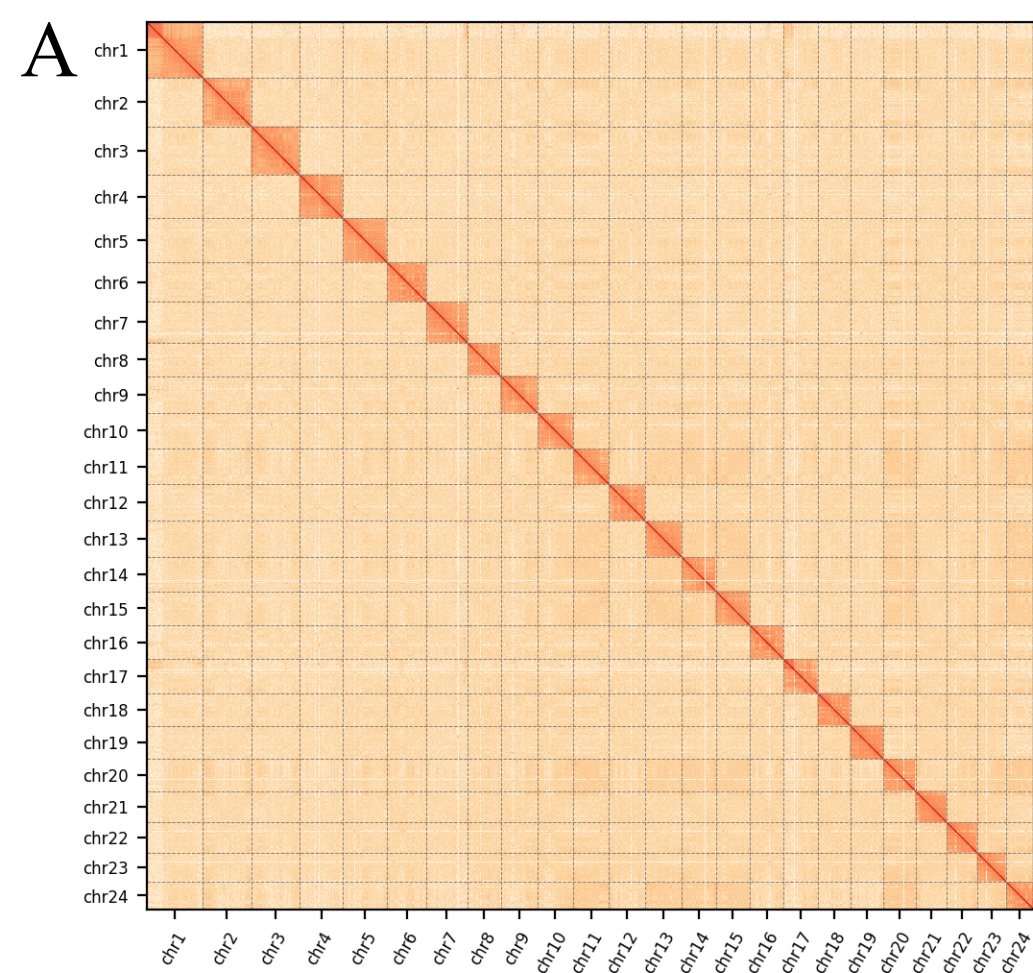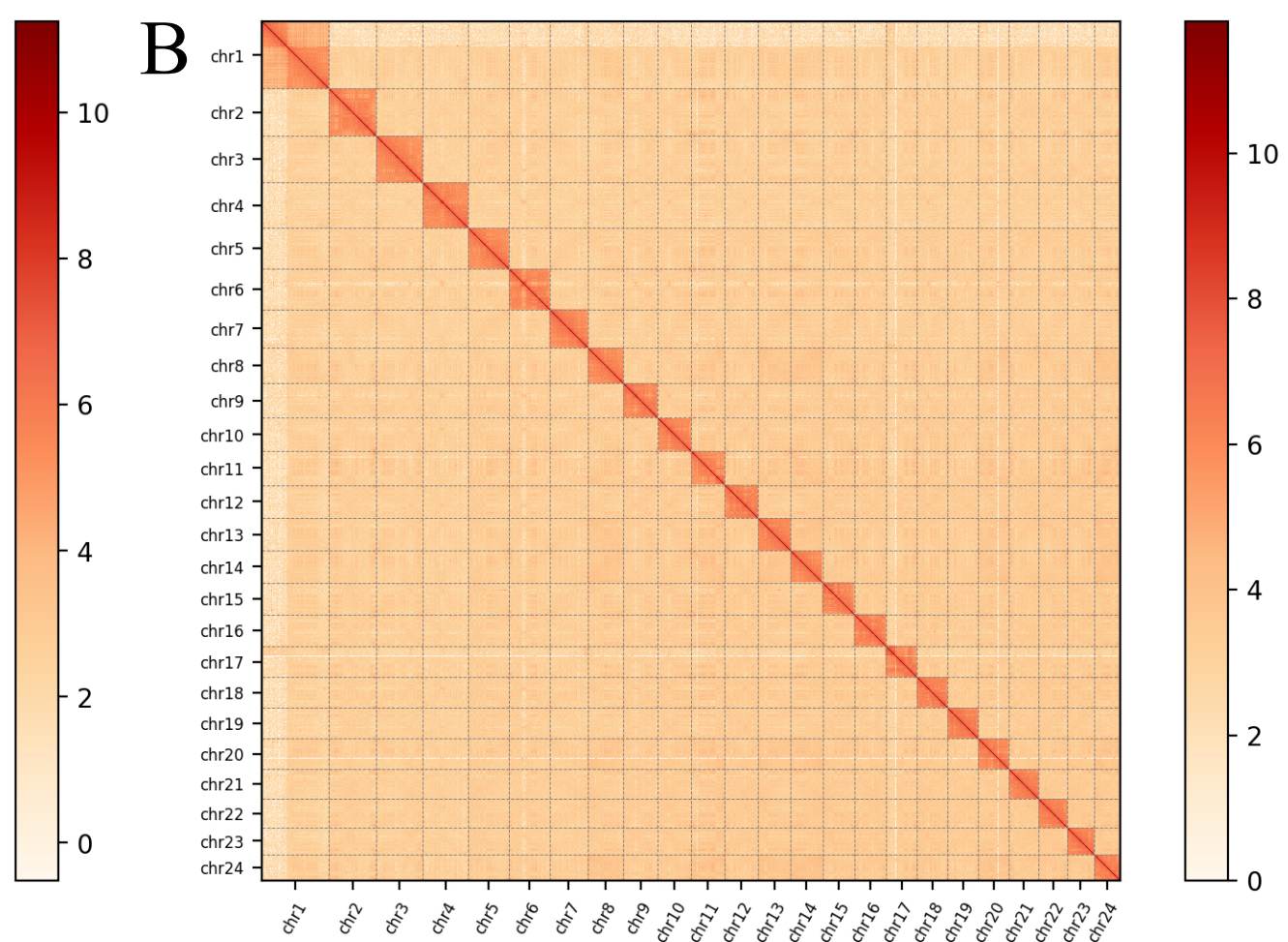

Supplement: Supplementary file 1 [file biomolecules-16-00083-s001.zip › Supplementary Files/Supplementary Figure 2.pdf]
